# Young people who inject drugs in India have high HIV incidence and behavioural risk: a cross‐sectional study

**DOI:** 10.1002/jia2.25287

**Published:** 2019-05-22

**Authors:** Lakshmi Ganapathi, Allison M McFall, Aylur K Srikrishnan, Muniratnam S Kumar, Santhanam Anand, Gregory M Lucas, Shruti H Mehta, Sion K Harris, Sunil S Solomon

**Affiliations:** ^1^ Division of Infectious Diseases Boston Children's Hospital and Harvard Medical School Boston MA USA; ^2^ Department of Epidemiology The Johns Hopkins Bloomberg School of Public Health Baltimore MD USA; ^3^ YR Gaitonde Center for AIDS Research and Education Chennai India; ^4^ Division of Infectious Diseases The Johns Hopkins University School of Medicine Baltimore MD USA; ^5^ Center for Adolescent Substance Abuse Research Boston Children's Hospital and Harvard Medical School Boston MA USA

**Keywords:** injection drug users, injection risks, sexual risks, emerging‐adult, young‐adult, HIV incidence

## Abstract

**Introduction:**

There are limited data on young people who inject drugs (PWID) from low‐ and middle‐income countries where injection drug use remains a key driver of new HIV infections. India has a diverse injection drug use epidemic and estimates suggest that at least half of PWID are ≤30 years of age. We compared injection and sexual risk behaviours and HIV incidence between younger and older PWID and characterized uptake of HIV testing and harm reduction services to inform targeted HIV prevention efforts.

**Methods:**

We analysed cross‐sectional data from 14,381 PWID recruited from cities in the Northeast and North/Central regions of India in 2013 using respondent driven sampling (RDS). We compared “emerging‐adult” (18 to 24 years, 26% of sample) and “young‐adult” PWID (25 to 30 years, 30% of sample) to older PWID (>30 years, 44% of sample) using logistic regression to evaluate factors associated with three recent risk behaviours: needle‐sharing, multiple sexual partners and unprotected sex. We estimated age‐stratified cross‐sectional HIV incidence using a validated multi‐assay algorithm.

**Results:**

Compared to older adults, emerging‐adults in the Northeastern states were significantly more likely to share needles (males adjusted odds ratio [aOR] 1.82; females aOR 2.29, *p *<* *0.01), have multiple sexual partners (males aOR 1.56; females aOR 3.75, *p *<* *0.01), and engage in unprotected sex (males aOR 2.29, *p *<* *0.01). In the North/Central states, young‐adult males were significantly more likely to needle‐share (aOR 1.23, *p *<* *0.05) while emerging‐adult males were significantly more likely to have multiple sexual partners (aOR 1.74, *p *<* *0.05). In both regions, emerging‐adults had the lowest HIV testing. Participation in harm reduction services was low across all age groups. Annual HIV incidence was higher in emerging‐ and young‐adult PWID in the North/Central region: emerging‐adults: 4.3% (95% confidence interval [CI] 3.0, 5.6); young‐adults: 4.9% (95% CI 3.7, 6.2); older adults: 2.1% (95% CI 1.4, 2.8).

**Conclusions:**

Higher HIV incidence and engagement in risky behaviours among younger PWID compared to older PWID, coupled with low utilization of harm reduction services highlight the importance of targeting this population in HIV programming. Age‐specific interventions focused on addressing the needs of young PWID are urgently needed to curb the HIV epidemic in this vulnerable population.

## 1 Introduction

A recent United Nations AIDS report highlights that young people are a key driver of the HIV epidemic 1, accounting for more than a third of new HIV infections globally, and yet the global HIV response, including national strategic plans, largely neglect addressing young people from most‐at‐risk populations (MARP) such as people who inject drugs (PWID)^2^. Some of the fastest growing HIV epidemics worldwide are being driven by injection drug use 3. In countries where injection drug use is a growing phenomenon, ongoing transmission of HIV is likely concentrated in young people who newly initiate drug use and are often difficult to engage in harm reduction efforts 3, 4, 5.

India has an estimated 2.2 million people living with HIV and an estimated 1.1 million PWID 6. Historically, injection drug use has been a major driver of the HIV epidemic in the Northeastern states given their close proximity to the “Golden Triangle” region of heroin production (Myanmar, Laos, Thailand and Vietnam)^7^, ^8^. The Northeast region is geographically isolated and comprised of tribal communities that are linguistically and culturally distinct from the rest of India 9. Injection drug use in this region has been influenced by a variety of geo‐political and social factors including civil unrest, under‐development and conservative social mores 10, 11, 12. More recent studies have also drawn attention to increasing rates of injection drug use leading to burgeoning HIV epidemics in cities in the North, Northwest and Central Indian states 13, 14, 15.

As the Northeast has long been viewed as the “epicentre” of injection drug use in India and a high‐HIV prevalence region, HIV prevention efforts in this region have been underway since the mid‐1990s, with uniformly available harm reduction and HIV testing and treatment services central to these efforts. In contrast, there is variability in the distribution and density of such services in the rest of India, particularly in states where rising injection drug use is a more recent phenomenon (Table S1), in part because they have historically been considered low‐HIV prevalence states 16, 17, 18, 19. For example, Uttar Pradesh, the state with the largest population, has fewer HIV testing and antiretroviral therapy (ART) centres than similarly sized high‐HIV prevalence states 19, 20. As such, even if harm reduction and HIV testing and treatment services exist in states with new injection drug use epidemics, whether these services sufficiently cover the population at risk is unknown since accurate size estimates of PWID populations are still being collected 20. Nevertheless, national strategic plans (NSP) for MARP in India have embraced the UNAIDS 90‐90‐90 targets and established goals for participation in harm reduction services such as syringe services programmes (SSP) and opioid agonist therapy (OAT) for PWID 20, 21.

While estimates of young PWID in India vary and have primarily been obtained from convenience samples, large bio‐behavioural surveys sampling approximately 20,000 PWID across cities in India have identified those aged 30 years and under as comprising at least half of the surveyed population 22. Although these surveys document high‐risk injection and sexual risk behaviours among PWID 22, few provide insight into age‐related differences in HIV burden and risky behaviours. Furthermore, while these surveys note PWID participation in SSP and OAT as being well below NSP goals of 80% and 20% respectively in several states 22, little is known about the degree to which younger PWID access existing harm reduction services.

We previously described high HIV burden in a large cross‐sectional study of PWID across 15 Indian cities 13. The objectives of the current analysis are: (a) to compare substance use and psychosocial risk behaviours, harm reduction service utilization, and HIV incidence among younger and older PWID and (b) to identify factors associated with recent injection and sexual risk behaviours and evaluate whether these factors vary among PWID of different ages. Specifically, we sought to understand if younger PWID between the ages of 18 to 24 years had greater behavioural risk in Northeast and North/Central India, two regions that are epicentres of India's injection drug use epidemic 23, 24, 25, 26. We undertook this study recognizing the need for age‐disaggregated data from low‐and‐middle‐income countries such as India that may help to tailor efforts to reach this vulnerable population and inform policies and health services that seek to curb rising injection drug use and growing HIV epidemics among PWID in parts of India.

## 2 Methods

### 2.1 Study design and population

We performed secondary data analysis on a subset of PWID (N = 14,381) recruited as part of a cross‐sectional baseline assessment in a cluster‐randomized trial (ClinicalTrials.gov Identifier NCT0168670) across 15 cities in the Northeastern and North/Central Indian states 13, 27. Participants were recruited between January and December 2013 in seven cities in Northeast (Aizawl, Churachandpur, Dimapur, Gangtok, Imphal, Lunglei and Moreh) and eight cities in North/Central India (Amritsar, Bhubaneshwar, Bilaspur, Chandigarh, Kanpur, Ludhiana, Mumbai and New Delhi) using respondent‐driven sampling (RDS), a chain referral strategy 28. In each city, we partnered with NGOs that provide services to PWID and conducted preliminary ethnographic work 13, 29, 30. Recruitment in each city was initiated with two or three “seeds” – individuals identified in the ethnographic phase as being well connected in their PWID communities. Each seed was given two hologram‐labelled referral coupons to recruit up to two network members (i.e. others they knew who injected drugs). Unique identification numbers on the coupons established recruiter‐recruit relationships. Eligible network members who first presented with a coupon completed the study assessments and were considered wave 1 of recruitment. They were then each given two referral coupons to recruit up to two members of their network. The next round of individuals recruited and enrolled were considered wave 2, and so on. Participants provided an electronically captured fingerprint to prevent duplicate enrolment. RDS performance characteristics were favourable, including recruitment of the target sample of 1000 PWID at all but one city (Moreh), a large number of recruitment waves (median 22 waves; range 12 to 50), short recruitment periods (median 135 days, range 52 to 200), low homophily for HIV status and achievement of equilibrium in all sites.

Eligibility criteria included age ≥18 years, providing informed consent, a valid RDS coupon (except for seeds) and self‐reported drug injection in the previous two years. Site coordinators screened participants who presented a valid coupon and queried injection behaviours in addition to performing a visual check for injection marks to ensure that participants were indeed PWID. Participants completed an interviewer‐administered electronic survey that included modules on socio‐demographic and substance use characteristics, HIV testing experience, sexual and injection risk behaviours, psycho‐social risks and use of harm reduction services.

Participants provided a blood sample for HIV testing following completion of the survey and were provided pre‐and post‐test counselling. HIV was diagnosed on site using three rapid tests, with western blot confirmation in cases where rapid tests were indeterminate 13. In HIV‐positive participants, absolute CD4 + cell count and HIV RNA level were quantified and recent HIV infection was characterized according to a multi‐assay algorithm 13 that has been validated for HIV subtype C, the predominant subtype in India 31. Ethical oversight was provided by the YR Gaitonde Centre for AIDS Research and Education and the Johns Hopkins Medical School institutional review boards.

### 2.2 Measures

#### 2.2.1 Age categories

We classified our population into “emerging‐adults” (ages 18 to 24), “young‐adults” (ages 25 to 30) and older PWID (ages > 30). In defining the ages of “emerging adults,” we were informed by the following: (i) Prior studies in Northeast and North India showing greater injection risk behaviours in this age group 32, 33; (ii) evidence of “emerging adulthood” as a distinct developmental stage characterized by greater identity exploration and consequently higher engagement in risk behaviours 34; and (iii) frameworks highlighting late adolescence (ages 18 to 19) and young adulthood (ages 20 to 24) as important stages in the life course wherein behaviours impacting later adult outcomes originate, presenting opportunities for intervention 35, 36. While the construct of emerging adulthood was first explored in high‐income countries, it has more recently been studied in youth in emerging economies such as India 37, 38, 39, 40, 41, 42.

We defined “older adults” as those >30 years based on the following: (i) The median age of PWID observed in nationally representative bio‐behavioural surveys 22; (ii) recently revised definitions of youth in India 43; and (iii) prior studies examining risk behaviours across age groups among PWID in Northeast India that have used similar definitions 32.

#### 2.2.2 Outcome measures

To examine the influence of age on recent HIV risk behaviours, we chose three behaviours as outcomes of interest: (i) Recent needle sharing, defined as either receiving or passing a needle in the prior six months; (ii) recent multiple sexual partners, defined as having two or more sexual partners in the prior six months; and (iii) recent unprotected sex, defined as self‐report of any unprotected sex in the prior six months among participants who reported any sex during this period (n = 7877). We chose these behaviours because of extensive literature highlighting the relationship between these behaviours and HIV transmission 44, 45, 46.

#### 2.2.3 Additional age cut‐offs

Although we defined the ages of “emerging adults” and “older adults” *a priori*, our data offered support for these age cut‐offs. We generated weighted proportion tables and Lowess plots by age and gender in each region for the three outcomes of interest (Figures [Link], [Link], [Link], [Link], [Link], [Link], [Link], [Link], [Link]). The proportion tables and Lowess plots for male and female PWID in the Northeast showed inflections in risk occurring at approximately age 25 years and subsequently between approximately twenty‐eight and thirty one years across most risk behaviours. In the North/Central region, we did not generate these data for female PWID given the small number (n = 56 [0.7%]). However, we noted inflections in risk for male PWID occurring at approximately 40 years of age for some risk behaviours such as needle sharing. Although we retained our original age category definitions for comparison of HIV prevalence and incidence by age group, we conducted sensitivity analyses using additional age cut‐offs as guided by the proportion tables and Lowess plots in our analyses of correlates of recent risk behaviours.

#### 2.2.4 Descriptive characteristics

We compared the following characteristics across age groups: (a) demographics: marital status, education, employment and housing security; (b) substance use: lifetime history of drugs injected, non‐injection drug use in the previous six months, and severity of alcohol use (measured using AUDIT 47); (c) psychosocial risks: incarceration in the previous six months and experience of social support (measured using the Medical Outcomes Study 48), and co‐existing depression (measured using the Patient Health Questionnaire (PHQ‐9)^49^); (d) health and harm reduction service utilization: self‐report of ever having received HIV testing and report of lifetime and recent participation in SSP and OAT in the previous six months; and (e) awareness of status among HIV positive PWID assessed as follows: participants who tested positive but reported either negative or unknown status were classified as being “unaware” while participants who had concordant testing and self‐report were classified as being “aware.”

### 2.3 Statistical methods

Data from RDS “seeds” were excluded. A composite weight including the RDS‐II (Volz‐Heckatorn) weight 50 and the relative population of PWID in each city derived from state‐level data 51 was applied to obtain population‐level estimates for each region. Unweighted estimates are provided in Table S2. In bivariate analyses, we compared the distribution of variables (all of which were categorical) across age groups using the chi‐square test. Given the large sample size, even small differences were statistically significant. Therefore, we used a value of > 0.2 on the Cramer's V, a measure of correlation between nominal variables ranging from 0.0 to 1.0, to determine associations with at least a moderate effect size.

The three outcomes of interest were compared across age groups using logistic regression (LR) modelling with generalized estimating equations (GEE) to account for clustering by site and recruitment by “seeds,” adjusting for relevant confounders. In addition to age category, all variables that were significantly associated with the outcomes of interest in univariate analyses (*p *<* *0.1) were entered into the multivariate models in a stepwise manner and retained if significant at a *p *<* *0.05. Our initial models used older adults (i.e. age > 30) as the comparison group. We performed sensitivity analyses with additional age cut‐offs in subsequent models to determine if age category predictors varied significantly.

All analyses were stratified by gender and geography (Northeast vs. North/Central), because our prior work has shown distinct regional differences in the injection drug use epidemic 13, 52. In the North/Central region, given the small number of female PWID and as regression models with and without women yielded similar results, we restricted models for this region to male PWID.

We applied the composite weight to obtain HIV population prevalence for each age group in both regions. As previously described 13, we estimated annualized HIV incidence (*I*) using the following equation: *I = w/nμ* where *w* is the number of HIV‐positive patients with recent infection by the multi‐assay algorithm, n is the number of HIV‐negative patients and *μ* is the window period in years (0.56), which was based on optimization for HIV serotype C 53. Incidence estimates were not weighted as these were formula‐derived cross‐sectional estimates, and there is no accepted method for weighting such estimates in RDS samples 53. Incidence and prevalence estimates across age groups were compared using the Kruskal‐Wallis test. All analyses were conducted using SPSS version 24.0 (IBM technologies, Armonk, New York).

## 3 Results

### 3.1 Descriptive characteristics

#### 3.1.1 Demographics

In both regions, more than a quarter of PWID recruited were emerging‐adults and more than a quarter were young‐adults (Table 1). In both regions, compared to older PWID, a significantly greater proportion of emerging‐adult PWID reported being unmarried and unemployed (*p *<* *0.01, Cramer's V > 0.2). PWID across age groups were predominantly heterosexual. The median age of injection initiation for the overall cohort was 21 years (interquartile range [IQR]: 18 to 26). 12.6% and 16% of emerging‐adults in the Northeast and North/Central regions respectively had initiated injection drug use before the age of 16.

**Table 1:** Characteristics of younger and older PWID in Northeast and North/Central India, n = 14,409

Characteristics	Northeast (N = 6543)				North/Central (N = 7866)			
	18 to 24 years Emerging adults n = 1837 (28.1%)	25 to 30 years Young adults n = 2019 (30.9%)	>30 years Older adults n = 2687 (41.1%)	Cramer's V	18 to 24 years Emerging adults n = 1998 (25.4%)	25 to 30 years Young adults n = 2300 (29.2%)	>30 years Older adults n = 3568 (45.4%)	Cramer's V
Demographics								
Gender								
Male PWID, n (%)	1611 (87.7)	1677 (83.1)	2220 (82.6)		1987 (99.4)	2280 (99.2)	3542 (99.3)	
Female PWID, n (%)	226 (12.3)	342 (16.9)	467 (17.1)		11 (0.6)	19 (0.8)	26 (0.7)	
Median age (years)(IQR)	21 (20 to 23)	28 (26 to 29)	36 (33 to 40)		22 (20 to 23)	28 (26 to 30)	38 (35 to 45)	
Median age of initiation of injection (years) (IQR)	18 (17 to 19)	20 (18 to 24)	22 (18 to 29)		18 (17 to 22)	23 (19 to 25)	30 (23 to 35)	
Injection initiation < 16 years, n (%)	232 (12.6)	186 (9.2)	194 (7.2)		319 (16.0)	192 (8.3)	101 (2.8)	
Median years of duration of drug use (years) (IQR)	3 (1 to 5)	7 (3 to 10)	14 (8 to 19)		2 (1 to 5)	5 (2 to 8)	10 (4 to 15)	
Median monthly family income (rupees) (IQR)	20,000 (12,000 to 50,000)	20,000 (10,000 to 60,000)	20,000 (8000 to 80,000)		10,000 (6000 to 25,000)	8000 (5000 to 14,000)	6000 (4500 to 10,000)	
Sexual orientation								
Heterosexual, n (%)	1818 (99.0)	2005 (99.3)	2677 (99.6)	0.03**	1944 (97.3)	2236 (97.2)	3477 (97.4)	0.01
Homosexual/bisexual, n (%)	19 (1.0)	14 (0.7)	11 (0.4)		54 (2.7)	64 (2.8)	91 (2.6)	
Marital status								
Never married, n (%)	1181 (64.3)	657 (32.5)	366 (13.6)	0.44***	1631 (81.6)	1331 (57.9)	896 (25.1)	0.47***
Married/other, n (%)	656 (35.7)	1362 (67.5)	2321 (86.4)		368 (18.4)	969 (42.1)	2672 (74.9)	
Education								
Primary school/lower, n (%)	305 (16.6)	508 (25.2)	805 (30.0)	0.13***	981 (49.1)	1217 (52.9)	1836 (51.5)	0.03**
Secondary school/higher, n (%)	1532 (83.4)	1511 (74.8)	1882 (70.0)		1017 (50.9)	1082 (47.1)	1732 (48.5)	
Employment								
Student, n (%)	265 (16.5)	63 (3.8)	4 (0.2)	0.25***	121 (6.1)	8 (0.4)	0 (0.0%)	0.17***
Unemployed, n (%)	489 (30.4)	384 (22.9)	352 (15.8)		299 (15.0)	154 (6.8)	239 (6.7)	
Regular/seasonal, n (%)	856 (53.2)	1230 (73.3)	1865 (84.0)		1567 (78.9)	2118 (92.9)	3303 (93.3)	
Housing type								
Slum/homeless, n (%)	9 (0.5)	20 (1.0)	1 (0.1)	0.06***	414 (20.7)	642 (27.9)	927 (26.0)	0.06***
Stable housing, n (%)	1828 (99.5)	1999 (99.0)	2687 (99.9)		1585 (79.3)	1658 (72.1)	2641 (74.0)	
Substance/behavioural risks								
Lifetime drug use								
Heroin only, n (%)	350 (19.3)	498 (24.7)	859 (32.2)	0.16***	290 (14.8)	312 (13.8)	372 (10.7)	0.05***
Pharmaceuticals only, n (%)	995 (54.8)	756 (37.5)	760 (28.5)		1262 (64.5)	1380 (61.2)	2294 (65.9)	
Both, n (%)	470 (25.9)	763 (37.8)	1051 (39.4)		406 (20.7)	563 (25.0)	815 (23.4)	
Non‐injection drugs								
Yes, n (%)	840 (45.7)	893 (44.2)	1159 (43.1)	0.02	1438 (72.0)	1754 (76.3)	2684 (75.2)	0.04***
Recent needle‐sharing								
Yes, n (%)	490 (26.7)	467 (23.1)	476 (17.7)	0.09***	665 (33.3)	888 (38.6)	1250 (35.0)	0.04***
Injection with others								
Half of the time or more, n (%)	840 (52.5)	830 (48.9)	1024 (48.9)	0.03	756 (41.7)	823 (38.7)	1170 (36.2)	0.05***
Less than half of the time, n (%)	761 (47.5)	868 (51.1)	1072 (51.1)		1055 (58.3)	1304 (61.3)	2064 (63.8)	
Recent sexual partners								
2 or more, n (%)	256 (29.7)	312 (24.7)	225 (13.4)	0.17***	262 (34.0)	323 (28.5)	343 (18.9)	0.14***
Less than 2, n (%)	605 (70.3)	953 (75.3)	1448 (86.6)		508 (66.0)	812 (71.5)	1469 (81.1)	
Sex work								
Yes, n (%)	96 (5.3)	137 (6.8)	170 (6.3)	0.13	47 (2.4)	68 (3.0)	171 (4.8)	<0.01
Recent unprotected sex								
Yes, n (%)	728 (84.7)	1079 (85.3)	1411 (84.4)	0.01	587 (76.2)	876 (77.2)	1517 (83.7)	0.09***
Alcohol use^a^								
Hazardous/dependence, n (%)	865 (47.1)	981 (48.6)	1255 (46.7)	0.02	616 (30.8)	829 (36.1)	1534 (43.0)	0.10***
Psychosocial risks								
Recent incarceration								
Yes, n (%)	264 (14.4)	288 (14.3)	245 (9.1)	0.08***	174 (8.7)	210 (9.2)	218 (6.1)	0.05***
Social support^b^								
Low, n (%)	237 (13.1)	292 (14.5)	380 (14.1)	0.02	712 (36.9)	1001 (44.4)	1739 (49.5)	0.10***
Medium/high, n (%)	1577 (86.9)	1725 (85.5)	2307 (85.9)		1215 (63.1)	1254 (55.6)	1772 (50.5)	
Depression^c^								
Moderate/severe, n (%)	573 (31.2)	816 (40.4)	1133 (42.2)	0.10***	817 (40.9)	1008 (43.8)	1672 (46.9)	0.05***
Health/harm reduction services utilization								
HIV testing (ever)								
No, n (%)	1057 (57.5)	836 (41.4)	1044 (38.9)	0.16***	1334 (66.7)	1426 (62.0)	2282 (63.9)	0.04***
Awareness of status (HIV‐positive PWID)								
Yes, n (%)	110 (61.1)	249 (60.0)	660 (69.0)	0.09***	61 (18.2)	61 (12.4)	155 (23.9)	0.13***
Syringe services programme								
Never, n (%)	1323 (72.4)	1305 (64.8)	1908 (71.2)	0.07***	1266 (63.7)	1478 (64.7)	2335 (65.6)	0.02
Within six months, n (%)	429 (23.5)	537 (26.7)	542 (20.2)		669 (33.6)	729 (31.9)	1105 (31.0)	
Opioid agonist therapy								
Never, n (%)	1653 (90.8)	1714 (85.0)	2196 (81.9)	0.07***	1561 (78.5)	1789 (78.4)	2656 (74.8)	0.05***
Within six months, n (%)	96 (5.3%)	170 (8.4)	258 (9.6)		372 (18.7)	406 (17.8)	667 (18.8)	
HIV prevalence (%) (95% CI)^d^	9.8 (8.4 to 11.2)	20.6 (18.8 to 22.3)	35.6 (33.8 to 37.4)	*p *<* *0.01	16.8 (15.1 to 18.4)	21.5 (19.8 to 23.1)	18.2 (16.9 to 19.5)	*p *<* *0.01

Population characteristics calculated using a composite weight including the RDS‐II weight and the relative population of PWID in each city derived from state‐level data. IQR, interquartile range.

^a^Hazardous use defined by score at least 8 on Alcohol Use Disorder Identification Test (AUDIT) and dependence defined by AUDIT score at least 15; ^b^low support defined by a score < =10 on the Medical Outcomes Study (MOS) social support survey, moderate support defined by score 11 to 19, and good support defined by a score ≥ 20; ^c^moderate depression defined by score at least 10 on the Patient Health Questionnaire‐9 (PHQ‐9) and severe depression defined by PHQ score at least 15; ^d^prevalence estimates compared using the Kruskal‐Wallis test.

**p <* *0.05; ***p <* *0.01.

Regional differences in the demographics of PWID included differences in education, employment and housing security. In comparison to the North/Central region, a significantly higher proportion of PWID in the Northeast completed secondary school or higher (Northeast 75.3% vs. North/Central 48.7%, *p *<* *0.01, Cramer's V > 0.2). The vast majority of PWID in the North/Central region reported either regular or seasonal employment (with low wage occupations), while unemployment, particularly among emerging‐adult PWID, was significantly higher in the Northeast (Northeast 31.7% vs. North/Central 15.2%, *p *<* *0.01, Cramer's V > 0.2). There was virtually no housing insecurity reported by PWID in the Northeast, whereas nearly a quarter of PWID in the North/Central region reported either living in slums or being homeless.

#### 3.1.2 Substance use and other behavioural risk characteristics

Several characteristics were similar for PWID across both regions and age groups, including: moderate to high prevalence of recent non‐injection drug use (e.g. oral or inhalation drugs), and high hazardous alcohol use or dependence (a third to half of all PWID). However, there were unique trends in other risk behaviours in specific subpopulations by region or gender.

For example, while pharmaceuticals were the predominant drugs injected across both regions, there was higher heroin use in the Northeast. Recent needle‐sharing was significantly higher in the North/Central region as compared to the Northeast (North/Central 35.6% vs. Northeast 21.9%, *p *<* *0.01, Cramer's V > 0.2). Gender‐related trends were specific only to women in the Northeast, where a greater proportion reported engaging in sex work (females: 13.1%, males: 4.9%, *p *<* *0.01, Cramer's V < 0.2).

#### 3.1.3 Psychosocial risks

In both regions, a greater proportion of emerging‐adult and young‐adult PWID had recent incarceration whereas a greater proportion of older PWID had moderate or severe depression. PWID across all age groups in the Northeast reported good social support whereas more than a third of emerging‐adult and young‐adult PWID and nearly half of older PWID in the North/Central region expressed low social support.

#### 3.1.4 Health and harm reduction service utilization

HIV testing was significantly lower in the North/Central region as compared to the Northeast (North/Central 35.9% vs. Northeast 55.1%, *p *<* *0.01, Cramer's V > 0.2). A greater proportion of emerging‐adults in both regions had never received an HIV test, although the effect size for these associations was weak.

OAT and SSP utilization – whether assessed by lifetime or recent participation – was generally lower than current recommended targets across all age groups. Emerging‐adult PWID had lower utilization of OAT in the Northeast, as compared to older adults, although this association was weak. Whereas, SSP participation was higher in young‐adult PWID in the Northeast, as compared to older adults, although this association was also weak. SSP participation was comparable across age groups in the North/Central region.

### 3.2 Recent HIV risk behaviours by age group

#### 3.2.1 Needle sharing

In general emerging‐adults and young‐adults were more likely to report needle‐sharing, although the significance of these associations varied by region and gender (Table 2). Compared to older adults, emerging‐adults in the Northeast had significantly higher adjusted odds of needle sharing for both genders; however, only young‐adult men (but not young‐adult women) had significantly higher adjusted odds of needle sharing (Table 2). Findings from sensitivity analyses are reported in Table 5; notably, emerging‐adults in the Northeast had higher odds of needle sharing compared to both young‐adults and older adults.

**Table 2:** Correlates of recent needle sharing among 14,381 PWID across 15 Indian cities

	Needle‐sharing (last six months)					
	Northeast				North/Central	
	Males		Females		Males	
	OR (95% CI)	AOR (95% CI)	OR (95% CI)	AOR (95% CI)	OR (95% CI)	AOR (95% CI)
Age						
Emerging adults (18 to 24)	1.95 (1.71 to 2.24)	1.82 (1.45 to 2.27)***	2.04 (1.39 to 3.00)	2.29 (1.69 to 3.10)***	1.16 (1.03 to 1.31)	1.24 (0.93 to 1.65)
Young adults (25 to 30)	1.43 (1.25 to 1.63)	1.29 (1.10 to 1.53)***	1.22 (0.86 to 1.74)	1.24 (0.92 to 1.67)	1.21 (1.08 to 1.35)	1.23 (1.06 to 1.42)**
Older adults (>30)		Ref		Ref		Ref
Education						
Primary school or less	1.02 (0.88 to 1.17)	–	0.88 (0.64 to 1.21)	–	1.44 (1.31 to 1.58)	1.25 (1.05 to 1.50)**
Secondary school and above						Ref
Employment						
Unemployed/student	1.10 (0.97 to 1.24)	–	0.66 (0.48 to 0.91)	0.84 (0.54 to 1.30)	0.91 (0.78 to 1.06)	–
Regular/seasonal employment				Ref		
Housing						
Slum/homeless	–	–	–	–	1.36 (1.22 to 1.52)	1.06 (0.87 to 1.29)
Stable housing						Ref
Drugs injected						
Heroin only	–	0.93 (0.63 to 1.35)	–	0.83 (0.44 to 1.58)	–	0.50 (0.28 to 0.87)**
Pharmaceuticals only		0.75 (0.54 to 1.04)		0.51 (0.28 to 0.92)**		0.86 (0.64 to 1.17)
Both		Ref		Ref		Ref
Inject with multiple others						
Half of the time or more	1.36 (1.21 to 1.52)	1.35 (1.00 to 1.82)	1.52 (1.10 to 2.09)	1.52 (0.92 to 2.51)	1.80 (1.63 to 1.99)	1.80 (1.56 to 2.08)***
Less than half of the time		Ref		Ref		Ref
Injection frequency/day						
>1	4.47 (3.75 to 5.32)	2.04 (1.50 to 2.79)***	4.43 (3.05 to 6.43)	1.62 (0.93 to 2.82)	3.09 (2.80‐3.41)	2.29 (1.52 to 3.45)***
1/none		Ref		Ref		Ref
Alcohol use						
Hazardous use/dependence	1.06 (0.95 to 1.18)	–	1.67 (1.21 to 2.31)	1.27 (0.82 to 1.96)	1.11 (1.01 to 1.22)	1.08 (0.77 to 1.51)
None/non‐hazardous use				Ref		Ref
Depression						
Moderate/severe	1.21 (1.07 to 1.35)	1.19 (0.98 to 1.43)	1.07 (0.79 to 1.46)	–	1.65 (1.50 to 1.82)	1.43 (1.19 to 1.72)***
Mild/minimal		Ref				Ref
Incarceration						
Yes	1.29 (1.10 to 1.51)	1.22 (1.00 to 1.50)	2.35 (1.41 to 3.91)	1.69 (1.11 to 2.57)**	1.62 (1.39 to 1.87)	1.36 (1.11 to 1.66)***
No		Ref		Ref		Ref
Used SSP						
Yes	1.83 (1.63 to 2.05)	1.48 (1.23 to 1.79)***	1.52 (1.10 to 2.10)	0.87 (0.65 to 1.16)	0.96 (0.87 to 1.06)	–
No		Ref		Ref		
Used OAT (six months)						
Yes	0.82 (0.68 to 0.98)	0.77 (0.61 to 0.96)**	0.98 (0.52 to 1.85)	–	0.89 (0.79 to 1.01)	0.99 (0.61 to 1.63)
No		Ref				Ref

AOR indicates adjusted odds ratio; SSP, Syringe Services Programme, OAT, Opioid Agonist Therapy.

**p <* *0.05, ***p <* *0.01.

#### 3.2.2 Multiple sexual partners

With the exception of young‐adult women in the Northeast, emerging‐adults and young‐adults in both regions had significantly higher adjusted odds of having multiple recent sexual partners, when compared to older PWID (Table 3). Emerging‐adult women had nearly four times higher adjusted odds of having multiple sexual partners compared to older PWID. Sensitivity analyses did not alter these findings (Table 5).

**Table 3:** Correlates of having multiple recent sexual partners among 14,381 PWID across 15 Indian cities

	2 or more partners (last 6 months)					
	Northeast				North/Central	
	Males		Females		Males	
	OR (95% CI)	AOR (95% CI)	OR (95% CI)	AOR (95% CI)	OR (95% CI)	AOR (95% CI)
Age						
Emerging adults (18‐24)	1.47 (1.21‐1.78)	1.56 (1.17‐2.08)***	2.48 (1.50‐4.10)	3.75 (2.27‐6.17)***	1.55 (1.32‐1.82)	1.74 (1.09‐2.80)**
Young adults (25‐30)	1.71 (1.42‐2.06)	1.61 (1.34‐1.92)***	1.58 (0.97‐2.56)	1.78 (0.98‐3.25)	1.45 (1.24‐1.69)	1.50 (1.25‐1.81)***
Older adults (>30)		Ref		Ref		Ref
Sexual orientation						
Heterosexual	0.12 (0.06‐0.26)	0.18 (0.09‐0.39)***	0.81 (0.23‐2.83)	–	0.24 (0.18‐0.32)	0.30 (0.21‐0.43)***
Homosexual/bisexual		Ref				Ref
Marital status						
Unmarried	0.93 (0.80‐1.10)	–	1.18 (0.69‐2.03)	–	1.15 (1.01‐1.30)	1.01 (0.761.36)
Married						Ref
Education						
Primary school or less	0.93 (0.76 to.14)	–	0.88 (0.57‐1.36)	–	0.86 (0.76‐0.98)	0.86 (0.73‐1.02)
Secondary school and above						Ref
Employment						
Unemployed/student	0.94 (0.79‐1.12)	–	0.58 (0.37‐0.92)	0.71 (0.43‐1.17)	0.86 (0.69‐1.07)	
Regular/seasonal employment				Ref		
Alcohol use						
Hazardous use/dependence	2.44 (2.08‐2.86)	2.00 (1.43‐2.79)***	2.52 (1.67‐3.81)	1.57 (0.84‐2.91)	2.68 (2.35‐3.07)	2.52 (2.28‐2.77)***
None/non‐hazardous use		Ref		Ref		Ref
Incarceration (six months)						
Yes	2.77 (2.30‐3.33)	1.90 (1.44‐2.51)***	2.21 (1.21‐4.06)	1.38 (0.81‐2.34)	1.52 (1.26‐1.84)	1.38 (1.16‐1.66)***
No		Ref		Ref		Ref
Sex work						
Yes	5.24 (4.09‐6.71)	3.51 (2.58‐4.77)***	16.0 (10.1‐25.5)	15.2 (7.90‐29.1)***	4.99 (3.91‐6.35)	3.99 (2.59‐6.15)***
No		Ref				Ref
Depression						
Moderate/severe	2.27 (1.94‐2.65)	1.93 (1.49‐2.51)***	2.22 (1.48‐3.34)	2.34 (1.35‐4.05)***	1.16 (1.02‐1.32)	1.05 (0.92‐1.19)
Mild/minimal		Ref		Ref		Ref
Used OAT (6 months)						
Yes	1.03 (0.81‐1.32)	–	0.56 (0.20‐1.60)	–	1.03 (0.87‐1.20)	–
No						

AOR, adjusted odds ratio; OAT, Opioid Agonist Therapy.

**p <* *0.05, ***p <* *0.01.

#### 3.2.3 Unprotected sex

In the Northeast, emerging‐adult men had significantly higher adjusted odds of having unprotected sex compared to older PWID; however, emerging‐ and young‐adult women in the Northeast and men in the North/Central did not have higher adjusted odds of unprotected sex (Table 4). Sensitivity analyses did not alter these findings (Table 5).

**Table 4:** Correlates of recent unprotected sex among 7877 PWID across 15 Indian cities

	Unprotected sex (last six months)					
	Northeast				North/Central	
	Males		Females		Males	
	OR (95% CI)	AOR (95% CI)	OR (95% CI)	AOR (95% CI)	OR (95% CI)	AOR (95%CI)
Age						
Emerging adults (18 to 24)	1.26 (0.99 to 1.60)	2.29 (1.29 to 4.06)***	1.10 (0.55 to 2.23)	0.97 (0.46 to 2.03)	0.66 (0.54 to 0.80)	1.19 (0.81 to 1.74)
Young adults (25 to 30)	1.19 (0.96 to 1.47)	1.48 (0.90 to 2.43)	1.13 (0.61 to 2.10)	1.11 (0.73‐1.68)	0.75 (0.63 to 0.89)	1.01 (0.85 to 1.20)
Older adults (>30)		Ref		Ref		Ref
Sexual orientation						
Heterosexual	0.63 (0.14 to 2.76)	1.48 (0.90 to 2.43)	–	–	0.91 (0.58 to 1.43)	–
Homosexual/Bisexual		Ref				
Marital status						
Unmarried	0.51 (0.42 to 0.62)	0.36 (0.22 to 0.58)***	0.50 (0.22 to 1.16)	–	0.34 (0.29 to 0.40)	0.35 (0.27 to 0.44)***
Married		Ref				Ref
Education						
Primary school or less	1.83 (1.39 to 2.41)	1.56 (1.27 to 1.90)***	0.59 (0.34 to 1.02)	0.64 (0.36 to 1.14)	1.21 (1.04 to 1.42)	1.28 (1.10 to 1.50)***
Secondary school and above		Ref		Ref		Ref
Employment						
Unemployed/student	0.74 (0.60 to 0.92)	0.80 (0.61 to 1.04)	0.91 (0.52 to 1.57)	–	0.63 (0.49 to 0.82)	0.94 (0.75 to 1.17)
Regular/seasonal employment		Ref				Ref
Housing						
Slum/homeless	–	–	–	–	0.71 (0.59 to 0.85)	0.71 (0.44 to 1.16)
Stable housing						Ref
Alcohol use						
Hazardous use/dependence	1.91 (1.57 to 2.32)	1.75 (1.26 to 2.42)***	1.22 (0.65 to 2.27)	–	1.73 (1.49 to 2.02)	1.81 (1.30 to 2.53)***
None/non‐hazardous use		Ref	Ref			Ref
Incarceration (six months)						
Yes	1.97 (1.43 to 2.72)	1.67 (1.05 to 2.67)**	6.42 (0.86 to 47.7)	7.31 (0.85 to 62.9)	0.82 (0.64 to 1.04)	0.89 (0.65 to 1.23)
No		Ref		Ref		Ref
Sex work						
Yes	1.13 (0.76 to 1.67)	–	0.41 (0.23 to 0.73)	0.45 (0.22 to 0.93)**	0.98 (0.70 to 1.38)	–
No				Ref		
Number of sex partners						
≥2 partners	1.68 (1.31 to 2.15)	1.57 (1.16 to 2.14)***	0.53 (0.30 to 0.94)	0.74 (0.32 to 1.73)	0.90 (0.76 to 1.07)	–
1 partner		Ref		Ref		
Depression						
Moderate/severe	1.13 (0.94 to 1.38)	–	0.74 (0.43 to 1.27)	–	1.02 (0.87 to 1.19)	–
Mild/minimal						
Used OAT (six months)						
Yes	0.70 (0.53 to 0.92)	0.70 (0.51 to 0.98)**	4.39 (0.59 to 33.0)	–	0.76 (0.64 to 0.91)	0.73 (0.59 to 0.91)***
No		Ref				Ref

AOR, adjusted odds ratio; OAT, Opioid Agonist Therapy.

**p <* *0.05, ***p <* *0.01.

**Table 5:** Sensitivity analyses with varying age cut‐offs for age category correlates of recent risk behaviours

Northeast males		Northeast females		North/Central males	
Age (years)	AOR (95% CI)	Age (years)	AOR (95% CI)	Age (years)	AOR (95%CI)
Recent needle sharing					
18 to 24	1.80 (1.44 to 2.24)***	18 to 24	2.38 (1.82 to 3.12)***	18 to 24	1.20 (0.90 to 1.60)
25 to 29	1.33 (1.11 to 1.58)***	25 to 29	1.43 (1.03 to 1.97)**	25 to 29	1.19 (0.97 to 1.47)
≥30	Ref	≥30	Ref	≥30	Ref
18 to 24	1.40 (1.16 to 1.69)***	18 to 24	1.96 (1.44 to 2.68)***	18 to 24	1.03 (0.84 to 1.25)
25 to 30	Ref	25 to 30	Ref	25 to 30	Ref
18 to 24	1.35 (1.12 to 1.61)***	18 to 24	1.88 (1.29 to 2.73)***	18 to 24	1.02 (0.86 to 1.22)
25 to 29	Ref	25 to 29	Ref	25 to 29	Ref
				18 to 24	1.36 (0.96 to 1.92)
				25 to 39	1.31 (1.07 to 1.45)***
				≥40	Ref
	
				18 to 24	1.55 (1.12 to 2.15)***
				25 to 40	1.48 (1.23 to 1.78)***
				>40	Ref
	
				18 to 24	1.55 (1.12 to 2.15)***
				25 to 30	1.54 (1.22 to 1.95)***
				31 to 35	1.43 (1.18 to 1.72)***
				36 to 40	1.39 (1.22 to 1.58)***
				>40	Ref
Two or more recent sexual partners					
18 to 24	1.51 (1.12 to 2.05)***	18 to 24	3.85 (2.22 to 6.66)***	18 to 24	1.62 (1.05 to 2.49)**
25 to 29	1.64 (1.34 to 2.02)***	25 to 29	2.22 (0.91 to 5.43)	25 to 29	1.46 (1.22 to 1.74)***
≥30	Ref	≥30	Ref	≥30	Ref
18 to 24	0.97 (0.78 to 1.21)	18 to 24	2.08 (1.00 to 4.30)	18 to 24	1.17 (0.85 to 1.61)
25 to 30	Ref	25 to 30	Ref	25 to 30	Ref
18 to 24	0.92 (0.77 to 1.10)	18 to 24	1.71 (0.71 to 4.11)	18 to 24	1.14 (0.80 to 1.63)
25 to 29	Ref	25 to 29	Ref	25 to 29	Ref
		
				18 to 24	2.11 (1.29 to 3.44)***
				25 to 35	1.74 (1.46 to 2.06)***
				>35	Ref
Recent unprotected sex					
18 to 24	2.18 (1.32 to 3.59)***	18 to 24	0.96 (0.46 to 2.00)	18 to 24	1.18 (0.85 to 1.63)
25 to 29	1.43 (0.95 to 2.16)	25 to 29	1.01 (0.53 to 1.94)	25 to 29	1.01 (0.83 to 1.22)
≥30	Ref	≥30	Ref	≥30	Ref
18 to 24	1.75 (1.38 to 2.22)***	18 to 24	0.80 (0.43 to 1.50)	18 to 24	1.19 (0.87 to 1.61)
25 to 30	Ref	25 to 30	Ref	25 to 30	1.01 (0.85 to 1.20)
				31 to 39	1.01 (0.72 to 1.41)
18 to 24	1.69 (1.32 to 2.18)	18 to 24	0.85 (0.42 to 1.74)	≥40	Ref
25 to 29	Ref	25 to 29	Ref		

AOR, adjusted odds ratio.

**p <* *0.05, ***p <* *0.01.

### 3.3 Other correlates of recent risk behaviours

In both regions, recent incarceration, hazardous/dependent alcohol use and moderate/severe depression were in general associated with higher odds of recent risk behaviours whereas OAT participation was associated with lower odds (Tables 2, 3 to 4).

### 3.4 Burden of HIV by age group

**Figure 1:**
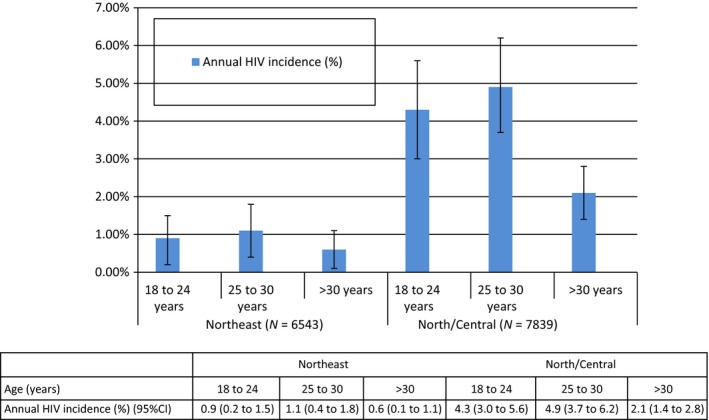
Annual HIV incidence among PWID in Northeast and North/Central India. Annual HIV incidence in emerging and young‐adult PWID compared to older PWID in the North/Central region was statistically significant.

In the North/Central region, annual HIV incidence was significantly higher among emerging‐ and young‐adults, who had the highest incidence of HIV in the overall sample (Figure 1). In the Northeast, while HIV incidence among emerging‐ and young‐adults was higher than in older adults, these differences did not achieve statistical significance (Figure 1). In the Northeast, HIV prevalence increased with age. However, in the North/Central region, HIV prevalence was highest among young‐adults (Table 1).

Among HIV‐positive PWID, a significantly greater proportion of PWID in the Northeast were aware of their status compared to those in the North/Central region (Northeast 65.6%, North/Central 18.8%, *p *<* *0.01, Cramer's V > 0.2). Although the effect size for these associations were weak, in both regions, a lower proportion of emerging and young‐adult HIV‐positive PWID compared to older HIV‐positive PWID were aware of their status.

## 4 Discussion

In this study, involving one of the largest cross‐sectional samples of PWID from India, we found that emerging‐ and young‐adults in North/Central India – the region with the most rapidly growing HIV and injection drug use epidemics – had higher HIV incidence than older PWID. Overall, HIV burden and risky behaviours were high and use of harm reduction services low among emerging‐ and young‐adult PWID across India. Furthermore, the lower levels of awareness of HIV status among emerging‐ and young‐adult HIV+ PWID compared to older PWID suggest that interventions targeting these vulnerable populations are critical for achieving the UNAIDS 90‐90‐90 targets in India.

A primary aim of this study was to understand if emerging‐adult PWID have greater behavioural risk, as has been previously reported in India (especially the Northeast) and other parts of the world 32, 33, 54, 55, 56. In this study, emerging‐adults did indeed have higher risk than both older adults and young‐adults for some behaviours in the Northeast – in particular needle‐sharing (in men and women) and unprotected sex (in men) – suggesting that this age construct has some explanatory value in this region for both genders. However, our results more generally revealed variability in age‐correlates of risk by region, gender and specific risk behaviour. In spite of this variability, a broader picture that emerges across both regions is one of generally elevated risk across most behaviours for PWID ≤ 30 years of age – that is, both emerging‐ and young‐adults – a finding reinforced by the higher HIV incidence in both of these groups in the North/Central region.

The greater relevance of the construct of emerging‐adulthood in the Northeast, as compared to the North/Central region, may be explained by differences in the regional social context. Emerging‐adult men in the Northeast were more highly educated and experienced less housing insecurity but also had greater unemployment than men in the North/Central region. Previous studies suggest that the lack of employment opportunities in the Northeast – often combined with ongoing financial support from their families and relative lack of responsibilities – has created a “social vacuum” for young men, who fill it in part with drug use as part of their identity exploration 57, 58.

In the North/Central region, only young‐adults had higher odds than older adults for both needle sharing and having multiple sexual partners. In addition, elevated risk for needle sharing in this region extended beyond the ages we defined as young‐adults, which could in part be explained by sparsely available SSP in some cities. Injection drug use in the North/Central region may be shaped by a context of greater financial hardship and poverty. Emerging and young‐adult PWID in the North/Central region had considerably lower family income, lower educational status, greater housing insecurity and lower social support than those in the Northeast. While PWID in the North/Central region had higher employment, these were often low‐wage occupations (manual labourer, auto‐rickshaw driver, etc.). The lower socio‐economic status of PWID in the North/Central region may require pooling of resources such as needles to support injection behaviours, which could be another explanation for the persistence of needle sharing in older age groups.

Our findings regarding unprotected sex differ with those from a prior study in Northeast India, which found higher likelihood of condom use among emerging‐adult male PWID 32. We found that emerging‐adult PWID men in the Northeast had higher adjusted odds of unprotected sex. In the North/Central region, emerging‐ and young‐adults had lower odds for unprotected sex in the univariate analysis; however, after adjusting for marital status there were no differences between age groups, potentially because married men were less likely to use condoms consistently.

In contrast to emerging‐adult PWID men in the Northeast, emerging‐adult PWID women had similar adjusted odds of unprotected sex compared to other age groups. This similar risk may partly reflect universally low condom use among women of all age groups. In addition, sex work among women was independently associated with lower odds of unprotected sex. Greater risk of unprotected sex among emerging‐adult PWID women may be mitigated by higher condom use negotiation power among young female sex workers, although this question merits further research.

While a detailed exploration of all correlates of risk behaviours is beyond the scope of this manuscript, incarceration was significantly associated with increased risk across multiple behaviours. A greater proportion of emerging‐ and young‐adult PWID had experienced incarceration, suggesting that it may be an important structural determinant of risk behaviours. Although the response to injection drug use in India has historically been punitive, the benefits of shifting towards a harm reduction approach is highlighted by the experience of the Northeastern states which were the first to institute widespread HIV prevention and treatment efforts based on harm reduction principles.

Although earlier multi‐city studies in other countries 59 emphasized the influence of HIV seroprevalence on HIV incidence rates among PWID, more recent studies suggest that community viral suppression, regardless of HIV seroprevalence rate, is a stronger predictor of HIV incidence in high‐risk populations 60, 61. In this study and previous studies, we show better awareness of HIV status and greater community viral suppression among PWID in the Northeast compared to those in the North/Central region 19, 62. Despite higher HIV prevalence in the Northeast, the overall lower HIV incidence in this region, including among emerging‐ and young‐adult PWID, is likely a result of these factors. The HIV incidence data from the Northeast states should encourage expanding implementation of combination HIV prevention strategies to states in the North/Central region, where some cities (e.g. Kanpur) continue to have no available harm reduction services, such as OAT.

The low median age of injection initiation in our sample suggests that nearly half of PWID in these Indian cities start injecting as adolescents, a trend also described in other countries 63, 64, 65. Almost 15% of emerging‐adults in our sample had begun injecting by age 16. These findings highlight the importance of shifting HIV prevention initiatives “downwards” to prevent injection initiation in the first place, as well as to intervene in the youngest users early in their injection careers, including in adolescence. The need to understand the burden of injection drug use among adolescents in India is underscored by a recent Supreme Court directive instructing the government to obtain reliable estimates of drug use in adolescents 66.

Although we note sub‐optimal utilization of SSP and OAT across all age groups, low utilization of these services is particularly concerning for emerging‐ and young‐adult PWID given their higher risk behaviours. These findings are prescient given that India recently developed a National Adolescent Programme (NAP) stipulating that adolescents and young adults should have access to youth‐specific services, including confidential HIV testing and substance use treatment. However, significant barriers remain to actual implementation of this public sector programme to benefit young people in MARP.

First, current goals of OAT participation of 20% in PWID may be insufficient for emerging‐adult and young‐adult users, and goals specific to these age groups with higher risk behaviours are needed in India's national strategic plan. We also found that recent OAT participation was associated with lower odds of risk behaviours, which lends further support for expansion of OAT participation targets. Second, the requirements for parental consent for OAT and HIV testing if under 18 years of age 67 may be another barrier for adolescent PWID motivated to receive services. Further research is needed to assess barriers to participation in existing harm reduction services and to identify gaps in the availability of youth‐specific substance use treatment services for young PWID.

## 5 Limitations and Strengths

We have presented regional estimates and recognize that city‐to‐city measures may vary and should ultimately influence local responses. Extrapolation of our findings to young PWID across other Indian cities should be done with caution. Recent injection and sexual risk behaviours were self‐reported and subject to recall and social desirability biases. As our study is cross‐sectional, a causal association between risk behaviours and HIV infection cannot be established. In addition, the age categories we used may have masked potential trends within each category. Although the small number of female PWID recruited in the North/Central region reflects the epidemiology of injection drug use among women in India 68, we were limited by these low numbers in evaluating age‐specific differences in risk behaviours. The low number of incident infections in the Northeast also restricted our power to examine age‐specific differences in incidence.

There is ample research highlighting the influence of network characteristics on engagement in high‐risk behaviours by PWID 44, 69, 70, 71, 72. We have previously described the role of network size on risk behaviours of PWID recruited in this cross‐sectional cohort 73. However, for this study we did not collect data on other network characteristics – such as demographics or risk behaviours of network members – and therefore were unable to evaluate the role such characteristics have on risk behaviours.

Our study has notable strengths including the systematic sampling methodology and the analytic weighting of estimates. Our analysis is among the first to provide age‐disaggregated data on risk behaviours and the HIV burden among emerging‐ and young‐adult PWID in both Northeast and North/Central India at such scale given our large sample size. Although our findings are derived from data collected in 2013, they remain relevant for several reasons: first, there is limited age‐disaggregated data focusing on HIV prevalence, incidence and risk taking behaviours among PWID in India. Second, most of the data that does exist is from the Northeastern states and in small samples. We have systematically collected data from PWID across India reflecting data from emerging drug use epidemics in North and Central India. The regional variability demonstrated in this data is also of importance for programme development. Third, as youth‐specific services are implemented, our data can serve as an important baseline to evaluate changes in risk behaviours as well as HIV burden among younger PWID in future studies.

## 6 Conclusions

Our findings reveal greater injection and sexual risk behaviours among emerging‐ and young‐adult PWID in two regions of India, which are epicentres of the country's injection drug use epidemic. These findings, juxtaposed with the low median age of injection initiation, may help to explain the high HIV burden in emerging‐ and young‐adults in both regions and the higher HIV incidence in these age groups in North/Central India. Our findings highlight an urgent need to address these younger PWID separately and specifically in policies and through development of targeted interventions and services.

Emerging‐ and young‐adult PWID in North/Central India in particular constitute a critical sub‐population driving the HIV epidemic. Interventions for young PWID in this region and across India should extend beyond generic “youth‐friendly” services to involve rapid scale‐up of novel age‐, region‐ and gender‐specific services. An agenda for addressing the needs of young PWID in India should involve identifying and ameliorating the structural, demographic and psycho‐social drivers of injection initiation and engagement in high‐risk behaviours; conducting a rapid assessment to obtain reliable size estimates of adolescent PWID; revising consent laws for HIV testing and OAT receipt among adolescent PWID; establishing ambitious targets for participation in harm reduction services by young PWID; and addressing barriers unique to young PWID for participation in such services.

## Competing interests

The authors declare that they have no competing interests.

## Authors’ contributions

A.M.M, A.K.S, M.S.K, S.A., G.M.L, S.H.M and S.S.S performed the research. L.G., S.K.H, S.S.S, A.M.M, G.M.L and S.H.M designed the research study. L.G. and S.K.H analysed the data. L.G wrote the paper. A.M.M., G.M.L., S.H.M., S.S.S. and S.K.H. provided key edits to the paper.

## Supporting information


**Figure S1.** Recent needle sharing by age among male PWID in the Northeast (n = 5505).

**Figure S2.** Recent needle sharing by age among female PWID in the Northeast (n = 1038).

**Figure S3.** Recent needle sharing by age among male PWID in the North/Central (n = 7802).

**Figure S4.** 2 or more sexual partners by age among male PWID in the Northeast (n = 5508).

**Figure S5.** 2 or more sexual partners by age among female PWID in the Northeast (n = 1037).

**Figure S6.** 2 or more sexual partners by age among male PWID in the North/Central (n = 7808).

**Figure S7.** Recent unprotected sex by age among male PWID in the Northeast (n = 3229)^†^.

**Figure S8.** Recent unprotected sex by age among female PWID in the Northeast (n = 570)^†^.

**Figure S9.** Recent unprotected sex by age among male PWID in the North/Central (n = 3679)^†^.

**Table S1.** Availability of harm reduction and HIV testing and treatment services in selected study cities

**Table S2.** Characteristics of younger and older PWID in Northeast and North/Central India, n = 14,381
