# Supplementary material for: Young people who inject drugs in India have high HIV incidence and behavioural risk: a cross‐sectional study
Source: J Int AIDS Soc. 2019 May 22;22(5):e25287. doi: 10.1002/jia2.25287 (PMC6530044; doi:10.1002/jia2.25287)
Supplement: Supplementary file 1 — Figure S1. Recent needle sharing by age among male PWID in the Northeast (n = 5505). [file JIA2-22-e25287-s001.docx]

**Appendix Figure 1: Recent needle sharing by age among male PWID in the Northeast (n=5505)**

| Age (years) | Proportion of participants reporting recent needle sharing (%) |
| --- | --- |
| 18 | 25.6 |
| 19 | 28.4 |
| 20 | 19.2 |
| 21 | 21.6 |
| 22 | 24.9 |
| 23 | 31.6 |
| 24 | 33.9 |
| 25 | 28.7 |
| 26 | 27.9 |
| 27 | 28.6 |
| 28 | 25.2 |
| 29 | 21.7 |
| 30 | 15.6 |
| 31 | 23.9 |
| 32 | 17.7 |
| 33 | 25.0 |
| 34 | 11.4 |
| 35 | 15.9 |
| 36 | 20.8 |
| 37 | 11.6 |
| 38 | 13.3 |
| 39 | 9.0 |
| >=40 | 18.9 |

**
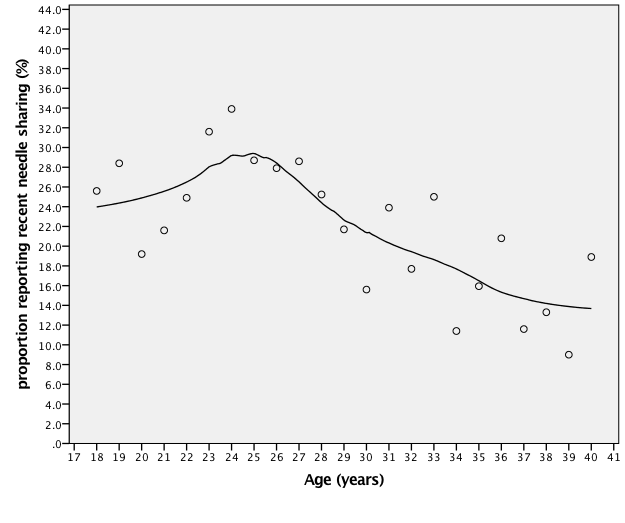
**
