# Supplementary material for: Young people who inject drugs in India have high HIV incidence and behavioural risk: a cross‐sectional study
Source: J Int AIDS Soc. 2019 May 22;22(5):e25287. doi: 10.1002/jia2.25287 (PMC6530044; doi:10.1002/jia2.25287)

**Appendix Figure 2: Recent needle sharing by age among female PWID in the Northeast (n=1038)**

| Age (years) | Proportion of participants reporting recent needle sharing (%) |
| --- | --- |
| 18 | 30.0 |
| 19 | 10.2 |
| 20 | 57.6 |
| 21 | 10.0 |
| 22 | 37.8 |
| 23 | 20.0 |
| 24 | 38.1 |
| 25 | 29.2 |
| 26 | 24.2 |
| 27 | 20.0 |
| 28 | 13.5 |
| 29 | 10.7 |
| 30 | 13.0 |
| 31 | 42.1 |
| 32 | 14.3 |
| 33 | 22.2 |
| 34 | 9.4 |
| 35 | 10.5 |
| 36 | 6.1 |
| 37 | 24.2 |
| 38 | 18.4 |
| 39 | 15.4 |
| >=40 | 23.3 |


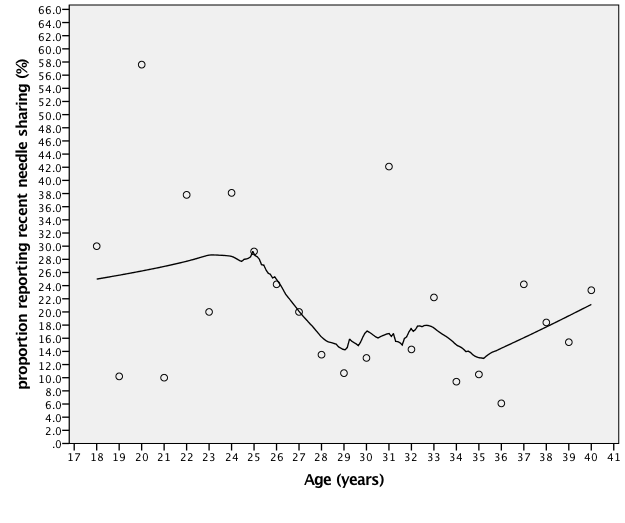

Supplement: Supplementary file 2 — Figure S2. Recent needle sharing by age among female PWID in the Northeast (n = 1038). [file JIA2-22-e25287-s002.docx]
