# Supplementary material for: Young people who inject drugs in India have high HIV incidence and behavioural risk: a cross‐sectional study
Source: J Int AIDS Soc. 2019 May 22;22(5):e25287. doi: 10.1002/jia2.25287 (PMC6530044; doi:10.1002/jia2.25287)

**Appendix Figure 3: Recent needle sharing by age among male PWID in the North/Central (n=7802)**

| Age (years) | Proportion of participants reporting recent needle sharing (%) |
| --- | --- |
| 18 | 41.0 |
| 19 | 18.1 |
| 20 | 38.4 |
| 21 | 31.5 |
| 22 | 40.5 |
| 23 | 30.4 |
| 24 | 30.5 |
| 25 | 40.5 |
| 26 | 27.0 |
| 27 | 30.8 |
| 28 | 43.8 |
| 29 | 26.4 |
| 30 | 44.3 |
| 31 | 24.1 |
| 32 | 38.1 |
| 33 | 24.2 |
| 34 | 35.2 |
| 35 | 41.3 |
| 36 | 28.5 |
| 37 | 34.8 |
| 38 | 50.0 |
| 39 | 21.2 |
| 40 | 38.3 |
| 41 | 51.1 |
| 42 | 39.5 |
| 43 | 21.5 |
| 44 | 28.6 |
| 45 | 37.5 |
| >=46 | 27.3 |


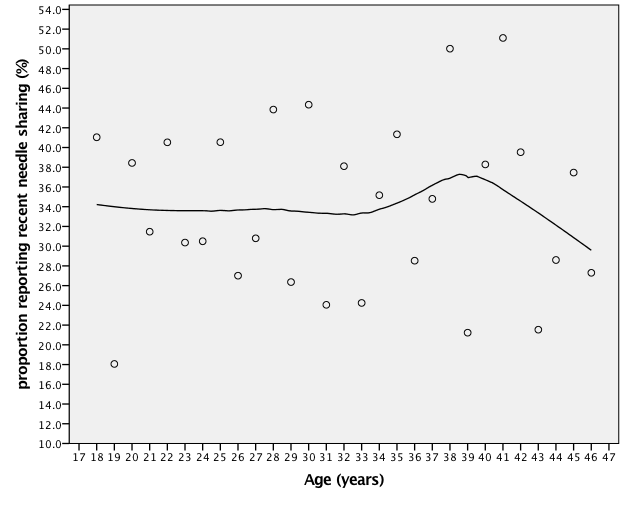

Supplement: Supplementary file 3 — Figure S3. Recent needle sharing by age among male PWID in the North/Central (n = 7802). [file JIA2-22-e25287-s003.docx]
