# Supplementary material for: Young people who inject drugs in India have high HIV incidence and behavioural risk: a cross‐sectional study
Source: J Int AIDS Soc. 2019 May 22;22(5):e25287. doi: 10.1002/jia2.25287 (PMC6530044; doi:10.1002/jia2.25287)

**Appendix figure 4: 2 or more sexual partners by age among male PWID in the Northeast (n=5508)**

| Age (years) | Proportion of participants reporting 2 or more recent sexual partners (%) |
| --- | --- |
| 18 | 3.4 |
| 19 | 15.1 |
| 20 | 18.5 |
| 21 | 12.6 |
| 22 | 17.9 |
| 23 | 17.3 |
| 24 | 13.6 |
| 25 | 16.1 |
| 26 | 15.2 |
| 27 | 20.9 |
| 28 | 16.4 |
| 29 | 16.7 |
| 30 | 14.3 |
| 31 | 7.5 |
| 32 | 13.2 |
| 33 | 5.1 |
| 34 | 2.4 |
| 35 | 7.1 |
| 36 | 7.7 |
| 37 | 10.7 |
| 38 | 6.1 |
| 39 | 30.5 |
| >=40 | 8.4 |


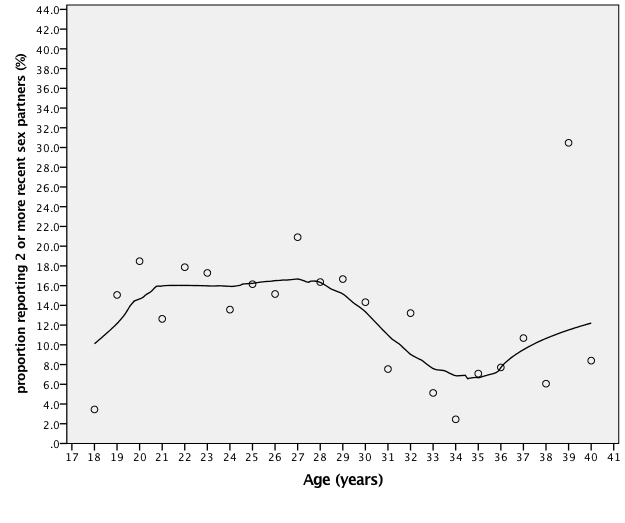

Supplement: Supplementary file 4 — Figure S4. 2 or more sexual partners by age among male PWID in the Northeast (n = 5508). [file JIA2-22-e25287-s004.docx]
