# Supplementary material for: Young people who inject drugs in India have high HIV incidence and behavioural risk: a cross‐sectional study
Source: J Int AIDS Soc. 2019 May 22;22(5):e25287. doi: 10.1002/jia2.25287 (PMC6530044; doi:10.1002/jia2.25287)

**Appendix figure 6: 2 or more sexual partners by age among male PWID in the North/Central (n=7808)**

| Age (years) | Proportion of participants reporting 2 or more recent sexual partners (%) |
| --- | --- |
| 18 | 10.1 |
| 19 | 12.4 |
| 20 | 15.3 |
| 21 | 17.0 |
| 22 | 13.3 |
| 23 | 11.2 |
| 24 | 11.5 |
| 25 | 14.6 |
| 26 | 16.5 |
| 27 | 15.5 |
| 28 | 14.7 |
| 29 | 17.9 |
| 30 | 11.0 |
| 31 | 14.2 |
| 32 | 14.3 |
| 33 | 11.3 |
| 34 | 7.4 |
| 35 | 14.3 |
| 36 | 9.3 |
| 37 | 6.0 |
| 38 | 9.2 |
| 39 | 24.1 |
| 40 | 8.4 |
| 41 | 17.3 |
| 42 | 5.1 |
| 43 | 6.1 |
| 44 | 16.3 |
| 45 | 6.2 |
| >=46 | 4.8 |


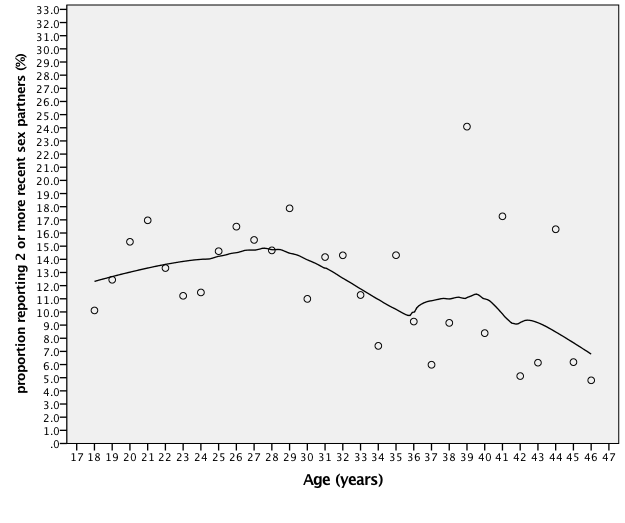

Supplement: Supplementary file 6 — Figure S6. 2 or more sexual partners by age among male PWID in the North/Central (n = 7808). [file JIA2-22-e25287-s006.docx]
