# Supplementary material for: Young people who inject drugs in India have high HIV incidence and behavioural risk: a cross‐sectional study
Source: J Int AIDS Soc. 2019 May 22;22(5):e25287. doi: 10.1002/jia2.25287 (PMC6530044; doi:10.1002/jia2.25287)
Supplement: Supplementary file 7 — Figure S7. Recent unprotected sex by age among male PWID in the Northeast (n = 3229)†. [file JIA2-22-e25287-s007.docx]

**Appendix Figure 7: Recent unprotected sex by age among male PWID in the Northeast (n=3229) †**

| Age (years) | Proportion of participants reporting recent unprotected sex (%) |
| --- | --- |
| 18 | 86.7 |
| 19 | 77.7 |
| 20 | 82.8 |
| 21 | 85.4 |
| 22 | 72.7 |
| 23 | 95.1 |
| 24 | 86.0 |
| 25 | 85.2 |
| 26 | 89.4 |
| 27 | 85.9 |
| 28 | 79.2 |
| 29 | 81.3 |
| 30 | 87.3 |
| 31 | 79.3 |
| 32 | 91.2 |
| 33 | 91.3 |
| 34 | 84.3 |
| 35 | 89.7 |
| 36 | 92.2 |
| 37 | 92.4 |
| 38 | 86.1 |
| 39 | 88.4 |
| >=40 | 75.2 |


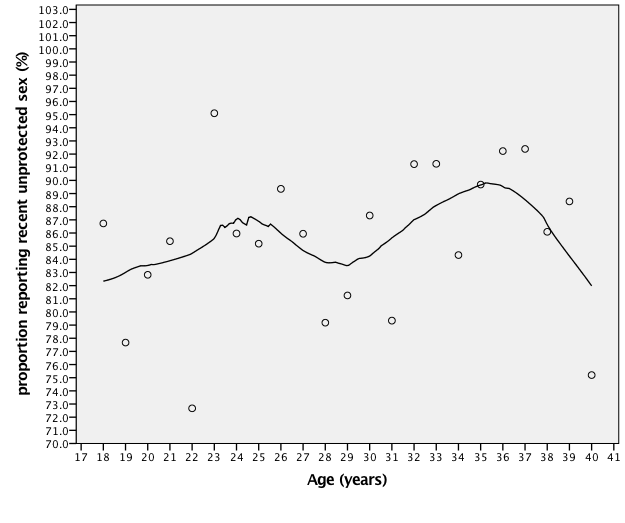


**† Includes PWID who reported vaginal/anal sex in the previous 6 months**
