# Supplementary material for: Young people who inject drugs in India have high HIV incidence and behavioural risk: a cross‐sectional study
Source: J Int AIDS Soc. 2019 May 22;22(5):e25287. doi: 10.1002/jia2.25287 (PMC6530044; doi:10.1002/jia2.25287)
Supplement: Supplementary file 8 — Figure S8. Recent unprotected sex by age among female PWID in the Northeast (n = 570)†. [file JIA2-22-e25287-s008.docx]

**Appendix Figure 8: Recent unprotected sex by age among female PWID in the Northeast (n=570) †**

| Age (years) | Proportion of participants reporting recent unprotected sex (%) |
| --- | --- |
| 18 | 100 |
| 19 | 100 |
| 20 | 100 |
| 21 | 66.7 |
| 22 | 64.0 |
| 23 | 100 |
| 24 | 91.7 |
| 25 | 100 |
| 26 | 84.6 |
| 27 | 93.2 |
| 28 | 76.6 |
| 29 | 80.0 |
| 30 | 82.6 |
| 31 | 63.6 |
| 32 | 87.5 |
| 33 | 100 |
| 34 | 41.7 |
| 35 | 87.8 |
| 36 | 74.2 |
| 37 | 88.2 |
| 38 | 86.7 |
| >=39 | 98.4 |


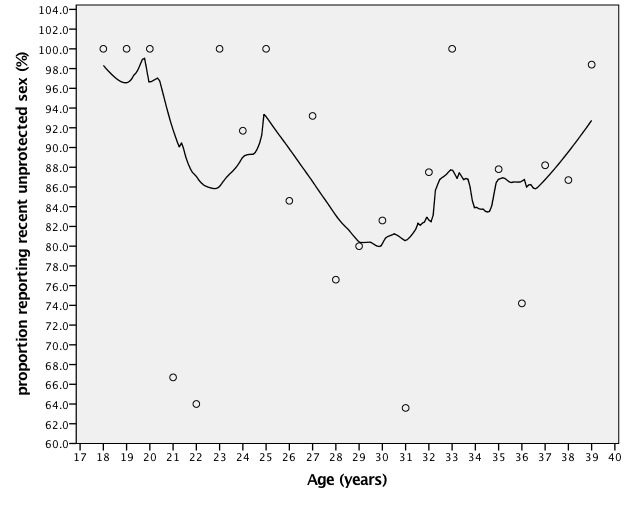


**† Includes PWID who reported vaginal/anal sex in the previous 6 months**
