# Supplementary material for: Young people who inject drugs in India have high HIV incidence and behavioural risk: a cross‐sectional study
Source: J Int AIDS Soc. 2019 May 22;22(5):e25287. doi: 10.1002/jia2.25287 (PMC6530044; doi:10.1002/jia2.25287)
Supplement: Supplementary file 9 — Figure S9. Recent unprotected sex by age among male PWID in the North/Central (n = 3679)†. [file JIA2-22-e25287-s009.docx]

**Appendix Figure 9: Recent unprotected sex by age among male PWID in the North/Central (n=3679) †**

| Age (years) | Proportion of participants reporting recent unprotected sex (%) |
| --- | --- |
| 18 | 81.5 |
| 19 | 78.3 |
| 20 | 68.2 |
| 21 | 73.2 |
| 22 | 81.1 |
| 23 | 84.0 |
| 24 | 74.1 |
| 25 | 75.5 |
| 26 | 81.8 |
| 27 | 77.5 |
| 28 | 75.1 |
| 29 | 68.6 |
| 30 | 80.1 |
| 31 | 68.2 |
| 32 | 79.8 |
| 33 | 80.0 |
| 34 | 72.6 |
| 35 | 87.3 |
| 36 | 90.5 |
| 37 | 79.7 |
| 38 | 90.4 |
| 39 | 91.4 |
| 40 | 84.4 |
| 41 | 75.6 |
| 42 | 88.2 |
| 43 | 81.1 |
| 44 | 88.9 |
| 45 | 93.2 |
| >=46 | 84.7 |


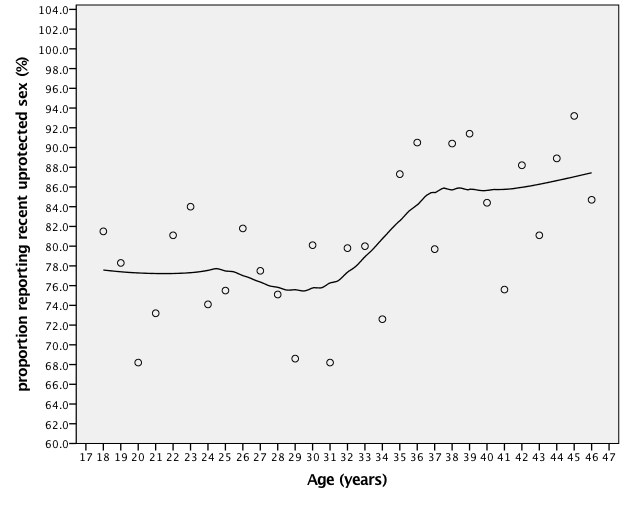


**† Includes PWID who reported vaginal/anal sex in the prior 6 months**
