# Supplementary material for: Young people who inject drugs in India have high HIV incidence and behavioural risk: a cross‐sectional study
Source: J Int AIDS Soc. 2019 May 22;22(5):e25287. doi: 10.1002/jia2.25287 (PMC6530044; doi:10.1002/jia2.25287)
Supplement: Supplementary file 10 — Table S1. Availability of harm reduction and HIV testing and treatment services in selected study cities [file JIA2-22-e25287-s010.docx]

**Appendix Table 1: Availability of harm reduction and HIV testing and treatment services in selected study cities**

| **Region (Northeast)** | **SSP** | **OAT** | **ART** | **ICTC** |
| --- | --- | --- | --- | --- |
| Imphal | 3 TI NGO points | 3 NGO points | 2 Government Centers | 5 locations |
| Dimapur | 3 TI NGO points | 2 NGO points and a Government center | 1 Government center | 3 locations |
| Aizawl | 5 TI NGO points | 4 NGO points | 2 Government centers | 5 locations and a mobile van |
| **Region (North/Central)** | **SSP** | **OAT** | **ART** | **ICTC** |
| Bilaspur | 2 NGO points | 2 Government sites | 1 Government center | 3 locations |
| Ludhiana | 2 NGO points | 1 Government center | 2 Government centers | 3 locations |
| Chandigarh | 3 NGO points | 3 NGO points | 2 Government centers | 4 locations |
| Amritsar | 2 NGO points | 3 sites | 1 Government center | 3 locations |
| Kanpur | 2 NGO points | None available | 1 Government center | 4 locations |
| New Delhi | 13 locations | 10 sites | 10 Government centers | 12 locations |

SSP – Syringe services program, OAT-Opioid agonist therapy, ART – Anti-retroviral therapy, ICTC –Integrated Counseling and Testing Center, NGO- non-governmental organization, TI – Targeted Intervention
