# Supplementary material for: Young people who inject drugs in India have high HIV incidence and behavioural risk: a cross‐sectional study
Source: J Int AIDS Soc. 2019 May 22;22(5):e25287. doi: 10.1002/jia2.25287 (PMC6530044; doi:10.1002/jia2.25287)
Supplement: Supplementary file 11 — Table S2. Characteristics of younger and older PWID in Northeast and North/Central India, n = 14,381 [file JIA2-22-e25287-s011.docx]

**Appendix Table 2. Characteristics of younger and older PWID in Northeast and North/Central India, n=14,381**

| **Characteristics** | **Northeast**  **(N= 6432)** | | | | **North/Central**  **(N=7949)** | | | |
| --- | --- | --- | --- | --- | --- | --- | --- | --- |
|  | 18-24 years  Emerging adults  n=1739(27.0%) | 25-30 years  Young adults  n=1966(30.6%) | >30 years  Older adults  n=2727(42.4%) | Cramer’s V | 18-24 years  Emerging adults  n=2039(25.7%) | 25-30 years  Young adults  n=2409(30.3%) | >30 years  Older adults  n=3501(44.0%) | Cramer’s V |
| **DEMOGRAPHICS** |  | | | |  | | | |
| **Gender** |  | | | |  | | | |
| Male PWID, n(%) | 1577 (90.7) | 1718 (87.4) | 2347 (86.1) |  | 2031 (99.6) | 2399 (99.6) | 3482 (99.5) |  |
| Female PWID, n(%) | 162 (9.3) | 248 (12.6) | 380 (13.9) |  | 8 (0.4) | 10 (0.4) | 19 (0.5) |  |
| Median age (IQR) | 21 (19-23) | 28 (26-29) | 36 (33-40) |  | 22 (20-23) | 28 (26-30) | 38 (34-44) |  |
| Median age of initiation)IQR) | 18 (16-19) | 20 (17-23) | 22 (18-28) |  | 18 (16-20) | 22 (18-25) | 28 (22-34) |  |
| Median duration of drug use (IQR) | 3 (2-5) | 8 (4-10) | 14 (9-19) |  | 3 (2-5) | 5 (3-9) | 10 (5-16) |  |
| Median monthly family income (rupees) (IQR) | 20,000(10,000-38,000) | 20,000(10,000-43,000) | 15,000(9,000-40,000) |  | 10,000(6,000-17,000) | 8,500(5,000-15,000) | 7,000(5,000-12,000) |  |
| **Sexual orientation** |  |  |  |  |  |  |  |  |
| Heterosexual, n(%) | 1724 (99.1) | 1947 (99.0) | 2716 (99.6) | 0.03 | 1967 (96.5) | 2334 (96.9) | 3415 (97.5) | 0.03 |
| Homosexual/Bisexual, n(%) | 15 (0.9) | 19 (1.0) | 11 (0.4) |  | 72 (3.5) | 75 (3.1) | 86 (2.5) |  |
| **Marital Status** |  |  |  |  |  |  |  |  |
| Unmarried, n(%) | 1118 (64.3) | 709 (36.1) | 422 (15.5) | 0.42^***^ | 1596 (78.3) | 1286 (53.4) | 871 (24.9) | 0.44^***^ |
| Married/ other, n(%) | 620 (35.7) | 1257 (63.9) | 2305 (84.5) |  | 443 (21.7) | 1123 (46.6) | 2630 (75.1) |  |
| **Education** |  |  |  |  |  |  |  |  |
| Primary school/lower,n(%) | 229 (13.2) | 393 (20.0) | 679 (24.9) | 0.12^***^ | 901 (44.2) | 1128 (46.8) | 1665 (47.6) | 0.03 |
| Secondary school/ higher,n(%) | 1510 (86.8) | 1573(80.0) | 2047 (75.1) |  | 1138 (55.8) | 1281 (53.2) | 1836 (52.4) |  |
| **Employment** |  |  |  |  |  |  |  |  |
| Student, n(%) | 409 (23.5) | 38 (1.9) | 7 (0.3) | 0.32^***^ | 120 (5.9) | 9 (0.4) | 0 (0.0) | 0.18^***^ |
| Unemployed, n(%) | 477 (27.4) | 426 (15.6) | 426 (15.6) |  | 304 (14.9) | 193 (8.0) | 264 (7.5) |  |
| Regular/seasonal, n(%) | 853 (49.1) | 1462 (74.4) | 2294 (84.1) |  | 1615 (79.2) | 2207 (91.6) | 3237 (92.5) |  |
| **Housing type** |  |  |  |  |  |  |  |  |
| Slum/homeless, n(%) | 6 (0.3) | 8 (0.4) | 2 (0.1) | 0.03 | 382 (18.7) | 609 (25.3) | 865 (24.7) | 0.07^***^ |
| Stable housing, n(%) | 1733 (99.7) | 1958 (99.6) | 2725 (99.9) |  | 1657 (81.3) | 1800 (74.7) | 2636 (75.3) |  |
| **SUBSTANCE/BEHAVIORAL RISKS** |  | | | |  | | | |
| **Lifetime drug use** |  |  |  |  |  |  |  |  |
| Heroin only, n(%) | 351(20.2) | 573 (29.1) | 1006(36.9) | 0.22^***^ | 331(16.2) | 450 (18.7) | 563(16.1) | 0.04^***^ |
| Pharmaceuticals only, n(%) | 919(52.9) | 598 (30.4) | 494 (18.1) |  | 1264(62.0) | 1396(67.9) | 2088(59.6) |  |
| Both, n(%) | 457(26.3) | 794 (40.3) | 1224(44.9) |  | 360(17.7) | 487(20.2) | 747(21.3) |  |
| **Non-injection drugs** |  |  |  |  |  |  |  |  |
| Yes, n(%) | 1111(63.9) | 1143(58.1) | 1530(56.1) | 0.07^***^ | 1460(71.6) | 1729(71.8) | 2364(67.5) | 0.05^***^ |
| **Recent needle-sharing** |  |  |  |  |  |  |  |  |
| Yes, n(%) | 737(42.4) | 677(34.4) | 744(27.3) | 0.13^***^ | 657(32.2) | 797(33.1) | 1019(29.1) | 0.04^***^ |
| **Injection with others** |  |  |  |  |  |  |  |  |
| Half of the time or more, n(%) | 889(55.0) | 814(45.6) | 1027(44.1) | 0.09^***^ | 828(45.2) | 877(39.9) | 1186(37.9) | 0.06^***^ |
| Less than half of the time, n(%) | 728(24.3) | 971(54.4) | 1302(55.9) |  | 1002(54.8) | 1319(60.1) | 1947(62.1) |  |
| **Recent sexual partners** |  |  |  |  |  |  |  |  |
| 2 or more, n(%) | 261(30.9) | 317(26.4) | 278(17.0) | 0.14^***^ | 325(36.2) | 360(27.5) | 379(19.1) | 0.15^***^ |
| Less than 2, n(%) | 585(69.1) | 882(73.6) | 1358(83.0) |  | 574(63.8) | 949 (72.5) | 1609(80.9) |  |
| **Recent sex work** |  |  |  |  |  |  |  |  |
| Yes, n(%) | 102(5.9) | 166(8.5) | 176(6.5) | 0.04^***^ | 66(3.2) | 90(3.7) | 142(4.1) | 0.02 |
| **Recent unprotected sex** |  |  |  |  |  |  |  |  |
| Yes, n(%) | 722(85.3) | 1016(84.7) | 1349(82.5) | 0.04 | 678(75.4) | 1021(78.0) | 1640(82.5) | 0.07^***^ |
| **Alcohol use**† |  |  |  |  |  |  |  |  |
| Hazardous/dependence, n(%) | 660(38.0) | 840(42.7) | 1063(39.0) | 0.04^***^ | 721(35.4) | 943(39.1) | 1580(45.1) | 0.08^***^ |
| **PSYCHOSOCIAL RISKS** |  | | | |  | | | |
| **Recent Incarceration** |  |  |  |  |  |  |  |  |
| Yes, n(%) | 244 (14.0) | 308(15.7) | 272(10.0) | 0.08^***^ | 236(11.6) | 290(12.1) | 311(8.9) | 0.05^***^ |
| **Social support**‡ |  |  |  |  |  |  |  |  |
| Low, n(%) | 231(13.3) | 310(15.8) | 523(19.2) | 0.07^***^ | 694(34.7) | 910(38.4) | 1541(44.5) | 0.08^***^ |
| Medium/High, n(%) | 1503(86.7) | 1652(84.2) | 2203(80.8) |  | 1306(65.3) | 1457(61.6) | 1922(55.5) |  |
| **Depression§** |  |  |  |  |  |  |  |  |
| Moderate/severe, n(%) | 489 (28.2) | 695(35.4) | 960(35.2) | 0.07^***^ | 753(36.9) | 1000(41.5) | 1522(43.5) | 0.05^***^ |
| **HEALTH/HARM REDUCTION SERVICES UTILIZATION** |  | | | |  | | | |
| **HIV testing (ever)** |  |  |  |  |  |  |  |  |
| No, n(%) | 1037(59.6) | 844(42.9) | 1019(35.1) | 0.18^***^ | 1276(62.6) | 1260(52.3) | 1755(50.1) | 0.10^***^ |
| **Awareness of status (HIV+PWID)** |  |  |  |  |  |  |  |  |
| Yes, n(%) | 76(58.0) | 205(54.2) | 609(61.6) | 0.07^**^ | 46(15.9) | 94(20.8) | 187(28.8) | 0.12^***^ |
| **Health services** |  |  |  |  |  |  |  |  |
| **Syringe Services Program** |  |  |  |  |  |  |  |  |
| Never, n(%) | 1059(61.2) | 1043(53.3) | 1488(54.8) | 0.05^***^ | 1259(62.2) | 1382(57.9) | 1967(56.4) | 0.04^***^ |
| Within 6 months, n(%) | 589(35.7) | 718(40.8) | 827(35.7) |  | 712(36.1) | 917(39.8) | 1338(40.5) |  |
| **Opioid Agonist Therapy** |  |  |  |  |  |  |  |  |
| Never, n(%) | 1569(90.5) | 1593(81.2) | 2029(74.6) | 0.10^***^ | 1598(79.3) | 1760(74.0) | 2390(68.8) | 0.07^***^ |
| Within 6 months, n(%) | 105(6.3) | 197(11.0) | 335(14.2) |  | 352(18.1) | 481(21.5) | 819(25.5) |  |
| **HIV prevalence (%)(95%CI)¶** | 7.5 (6.3-8.9) | 19.2(17.5-21.0) | 36.2(34.4-38.0) | <0.01 | 14.2(12.7-15.7) | 18.8(17.3-20.4) | 18.5(17.3-19.8) | <0.01 |

**Unweighted population characteristics; ^**^p<0.05;^***^p<0.01; IQR- interquartile range; †Hazardous use defined by score at least 8 on Alcohol Use Disorder Identification Test (AUDIT) and dependence defined by AUDIT score at least 15;** **‡Low support defined by a score ≤10 on the Medical Outcomes Study (MOS) social support survey, moderate support defined by score 11-19, and good support defined by a score ≥20; §Moderate depression defined by score at least 10 on the Patient Health Questionnaire-9 (PHQ-9) and severe depression defined by PHQ score at least 15;** **¶Prevalence estimates compared using the Kruskal-Wallis test.**
